# Central and rear-edge populations can be equally vulnerable to warming

**DOI:** 10.1038/ncomms10280

**Published:** 2015-12-22

**Authors:** Scott Bennett, Thomas Wernberg, Bijo Arackal Joy, Thibaut de Bettignies, Alexandra H. Campbell

**Affiliations:** 1 UWA Oceans Institute (M470) and School of Plant Biology, University of Western Australia, Crawley, Western Australia 6009, Australia; 2 Department of Environment and Agriculture, Curtin University, Bentley, Western Australia 6102, Australia; 3 Evolution and Ecology Research Centre, Centre for Marine Bio-Innovation, School of Biological, Earth and Environmental Sciences, University of New South Wales, Sydney, New South Wales 2052, Australia; 4 Sydney Institute of Marine Sciences, Chowder Bay, New South Wales 2088, Australia

## Abstract

Rear (warm) edge populations are often considered more susceptible to warming than central (cool) populations because of the warmer ambient temperatures they experience, but this overlooks the potential for local variation in thermal tolerances. Here we provide conceptual models illustrating how sensitivity to warming is affected throughout a species' geographical range for locally adapted and non-adapted populations. We test these models for a range-contracting seaweed using observations from a marine heatwave and a 12-month experiment, translocating seaweeds among central, present and historic range edge locations. Growth, reproductive development and survivorship display different temperature thresholds among central and rear-edge populations, but share a 2.5 °C anomaly threshold. Range contraction, therefore, reflects variation in local anomalies rather than differences in absolute temperatures. This demonstrates that warming sensitivity can be similar throughout a species geographical range and highlights the importance of incorporating local adaptation and acclimatization into climate change vulnerability assessments.

## Abstract (web summary)

Low latitude populations are often thought to be more vulnerable to climate change due to warmer ambient conditions. Here, Bennett *et al.* show that populations of seaweed from different areas of their range have distinct thermal-tolerance thresholds, but share remarkably similar thermal safety margins to warming.

## Graphical abstract

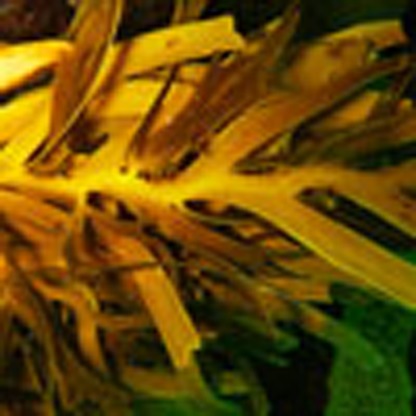

Climate change is altering species distributions across the globe^1^,^2^. A pressing challenge for climate change science is, therefore, to understand the sensitivity of organisms to the changing environment, which, given the global scale of the problem, ultimately requires extrapolation of local scale biological knowledge to broader spatial scales. Predicting species range shifts through species distribution modelling and by relating measured thermal-tolerance limits to species distributions has received a large research emphasis in recent years^3^,^4^,^5^. However, much of this work relies on thermal-tolerance estimates from a single climatic location^3^,^4^,^5^, and implicitly assumes conspecific populations living under different thermal regimes share a common thermal-tolerance breadth (that is, no local adaptation or acclimatization; Fig. 1a). This paradigm has a long history in ecology and biogeography^6^,^7^, and in the context of temperature thresholds implies that rear-edge (that is, low latitude and warm) populations have smaller thermal-safety margins (defined herein as the temperature buffer between an organisms upper thermal-tolerance limit and the maximum ambient temperatures it experiences) than central populations, making them more vulnerable to perturbation and species range contractions^6^ (Fig. 1a).

Along thermal gradients, however, selection pressure from the environment can lead to local adaptation and acclimatization of thermal-tolerance limits among populations^8^,^9^,^10^. Local adaptation can occur both when the spatial scale of the environmental gradient exceeds the dispersal distance of the organism^9^, and in areas of higher gene flow where post-settlement selection processes (that is, balanced polymorphism) can structure local tolerance limits^9^,^11^. Phenotypic acclimatization can also result in distinct thermal-tolerance limits among populations living under different climatic conditions, which can persist within a population for single-to-multiple generations after conditions change^12^,^13^. In the context of climate change and climate variability, both these processes imply that the absolute temperature threshold of a rear-edge population might be higher than that of the central population. Consequently, both rear-edge and central populations may have similar thermal-safety margins and sensitivity to climate impacts (Fig. 1b). Despite increasing evidence for the importance of local adaptation and acclimatization in multiple taxa^9^,^10^,^14^, its implications for differences in vulnerability among conspecific populations living under different climatic regimes have not been integrated into a spatially explicit framework of species sensitivity to climate change (but see refs 15, 16, Supplementary Fig. 1).

Here we apply ‘locally adapted' and ‘non-adapted' models of warming sensitivity to test whether a warm range edge (RE) population undergoing range contraction is indeed more vulnerable to warming and extreme events than central populations living under cooler climatic conditions. Specifically, we observed whether thermal-safety margins varied among populations, by assessing the absolute temperature and the maximum thermal-stress anomaly at which biological processes (for example, growth and reproduction) ceased to function. Thermal-stress anomalies (herein called stress anomalies) were defined as temperatures that exceed the climatologically (long-term average) warmest day of the year^17^, which in the context of the translocated seaweeds meant that temperatures from the recipient locations were compared to long-term averages from source locations. If populations do not have locally adapted thermal limits (that is, Fig. 1a), thermal-safety margins would differ throughout a species range and we would expect responses to high temperatures among populations to be best explained by variation in absolute maximum temperature. By contrast, if populations have locally adapted thermal limits, thermal-safety margins would remain similar throughout a species range and we would expect responses to high temperatures among populations to be better explained by stress anomalies, rather than the absolute temperature. Our results demonstrate that thermal-safety margins are remarkably similar for a habitat-forming seaweed from central and rear-edge populations, highlighting that conspecific populations can be equally sensitive to warming throughout a species geographical range.

## Results

### Regional climatic conditions during heatwave and experiment

**Figure 1:**
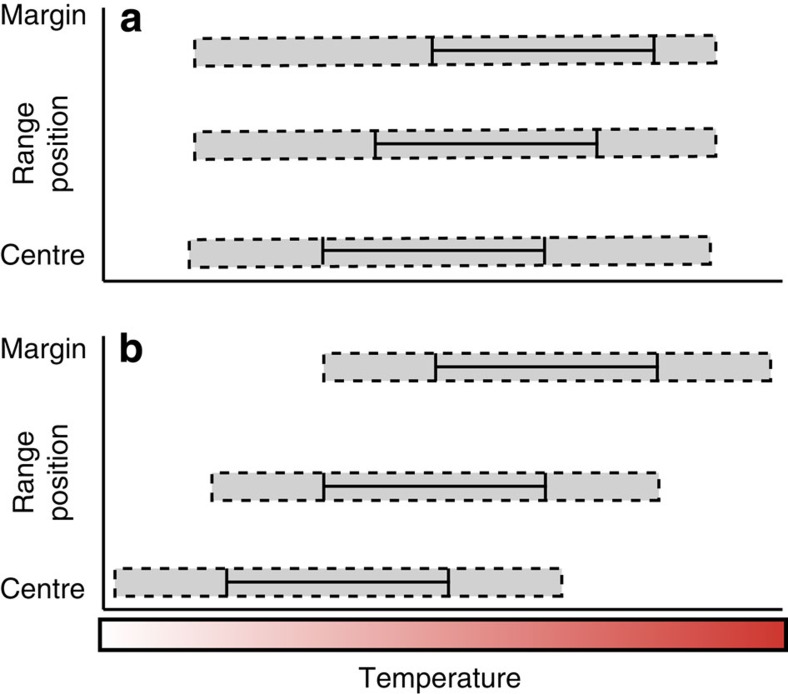
Model relationships between an organisms' experienced climatic range and its temperature tolerance breadth. Distance between the solid line and the edge of the dashed box represents the thermal-safety margin. The three solid lines per model represent the variation in climatic ranges throughout a species geographical range. Thermal-safety margins are influenced by whether there is (**a**) no local adaptation/acclimatization or (**b**) local adaptation/acclimatization in the upper thermal-tolerance threshold. Models are not mutually exclusive. (**a**) Experienced climatic ranges differ in absolute temperatures but have similar variability among populations. Thermal-tolerance breadth is the same, but thermal-safety margins decrease towards rear-edge populations. (**b**) Same conditions as **a**, but thermal-tolerance breadth changes with range position and thermal-safety margins remain constant among populations.

**Figure 2:**
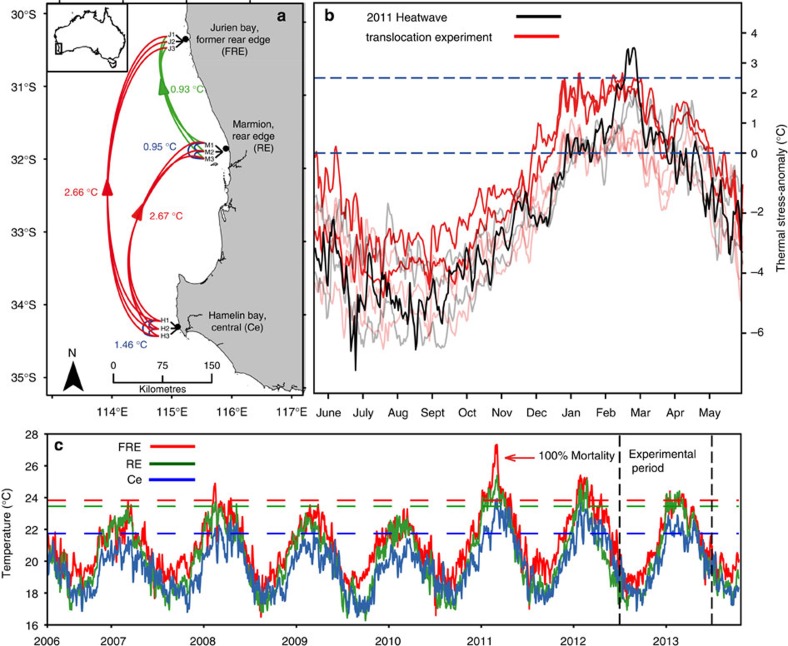
Map of study locations and climatic conditions experienced throughout the experiment. (**a**) Translocation source and recipient sites along west coast of Australia. Red lines represent translocations that experienced the highest thermal-stress anomalies, green and blue lines represent low stress anomalies and procedural controls. Numbers next to line specify the maximum stress anomaly experienced by each treatment (**b**) Stress anomalies experienced by *Scytothalia* in Jurien Bay at the FRE during the 2011 marine heatwave (black) and for the transplant and control populations during the experimental period (red). Bold lines illustrate the stress anomalies, where severe impacts to *Scytothalia* were observed. Dull lines represent the stress anomalies of locations and treatments where no impact was observed. Dashed lines illustrate 0 and 2.5 °C stress anomalies. Note that impacts only occurred where stress anomalies reached 2.5 °C. (**c**) Time series of mean daily temperatures in the three study locations. Dashed lines illustrate the maximum summer temperature for the three locations (averaged from 2006 to 2013).

During Austral summer of 2011, an extreme marine heatwave caused anomalous ocean temperatures (that is, temperatures exceeding anything recorded in the past 140 years) across 1,000 km of the west coast of Australia's Great Southern Reef^18^,^19^,^20^. *Scytothalia dorycarpa,* a dominant habitat-forming seaweed, underwent a ∼100 km range contraction from its former warm RE (FRE) in Jurien Bay (30°S) to Lancelin (31°S), where only a few scattered individuals remained^21^. Despite the broad spatial extent of the heatwave, higher latitude populations were unaffected by the event, suggesting that areas where *Scytothalia* still forms canopies, such as the new functional RE (Marmion, 32°S; Fig. 2) and central populations (Ce, that is, Hamelin Bay, 34°S) may have had larger thermal-safety margins than FRE populations, consistent with Fig. 1a.

To test whether variation in the thermal-safety margins predisposed FRE populations to range contraction, we simulated heatwave conditions by translocating *Scytothalia* from Ce to RE, from Ce to FRE and from RE to FRE (Fig. 2). Throughout the experiment, RE and FRE locations shared the highest recorded temperature of 24.4 °C (ca. 23.2 °C in Ce), representing the maximum stress anomaly of 2.7 °C for both Ce to RE and Ce to FRE transplants, relative to their reefs of origin (Table 1). The lowest maximum stress anomalies (0.9 °C) were recorded for RE to RE and RE to FRE transplants. Stress anomalies experienced by Ce to RE and Ce to FRE transplants were 0.5–0.7 °C above the highest stress anomalies experienced during the 2011 heatwave in Ce and RE locations, where no impacts were observed, and 0.9 °C below the maximum stress anomaly in the FRE, where *Scytothalia* suffered 100% mortality^21^ (Table 1, Fig. 2).

### Growth patterns of central and rear-edge populations

**Figure 3:**
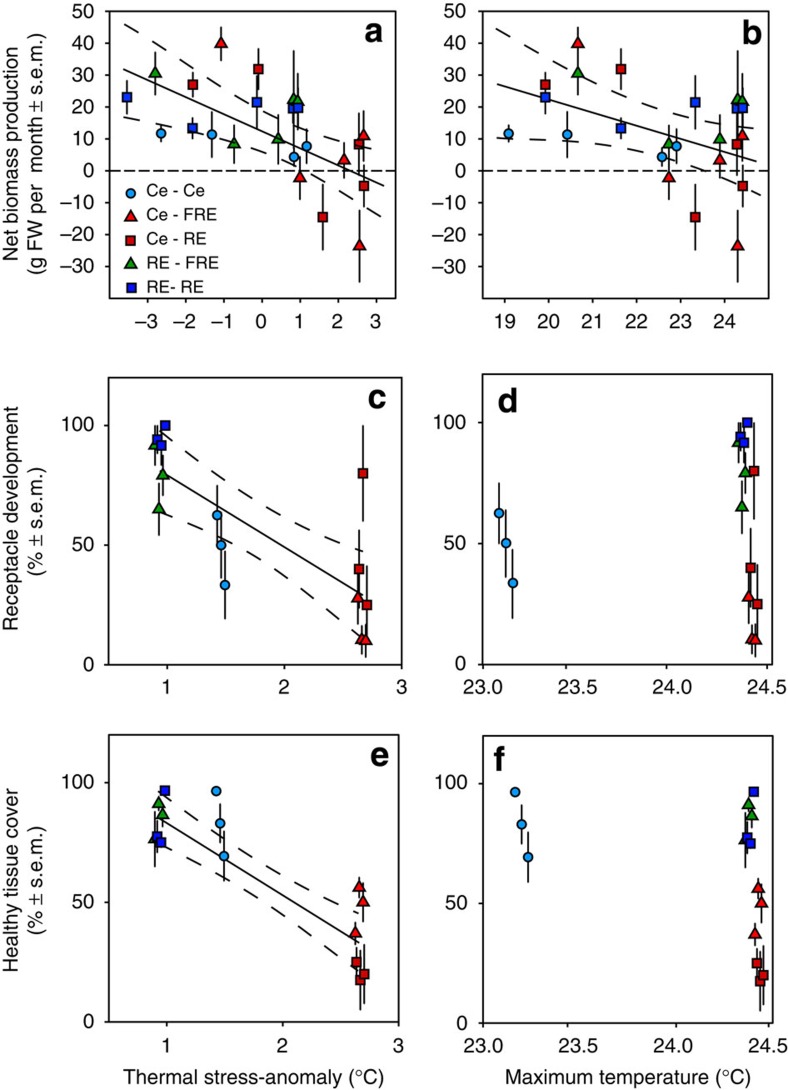
Comparison of physiological performance (mean±s.e.m.) in response to thermal-stress anomalies and the maximum temperatures. (**a**,**b**) Illustration of *Scytothalia* growth rates from September 2012–May 2013, in response to stress anomalies and the maximum temperatures, respectively. (**c**,**d**) Illustration of the proportion of individuals with receptacles at the onset of the reproductive season. (**e**,**f**) Illustration of the proportion of thallus area without visible putative disease symptoms. Significant linear models are marked with a regression line (solid) and 95% confidence intervals (dashed). Models are based on *n*=75 replicates for **a**,**b** and *n*=15 replicates for **c**–**f**. The shape and colour of points represents the five experimental treatments.

Differences among stress anomalies best explained the growth patterns with net biomass loss occurring once stress anomalies exceeded 2.5 °C (Fig. 3a, Table 2). When observed across the entire year, growth rates also declined in response to increasing local temperatures (Fig. 3b, Table 2). This pattern was driven by high growth rates within all experimental populations during cooler months and variable growth rates among treatments during the warmest months. Importantly, during the warmest period of the year, growth rates differed among treatments in response to the maximum stress anomalies but showed no response to the maximum temperatures *per se* (Table 2). This pattern resulted from sustained positive growth rates by RE to RE and RE to FRE transplants throughout summer months, where they experienced relatively small stress anomalies compared to Ce to RE and Ce to FRE transplants, despite growing under the same temperature conditions (Table 1, Supplementary Fig. 2).

### Reproductive development

**Figure 4:**
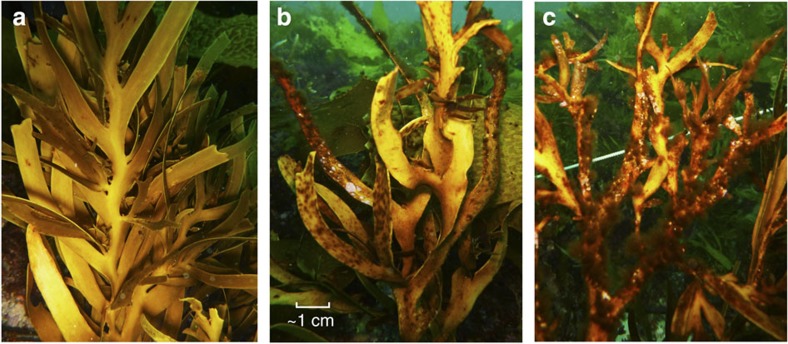
Impact of thermal-stress anomalies on *Scytothalia* transplants experiencing low and high thermal-stress anomalies. Thallus condition was initially healthy under low stress anomalies (that is, **a**). Under high stress anomalies thallus deterioration began with brown spotting and other putative disease symptoms (that is, **b**) before developing into high epibiont cover (**c**). Note that receptacles remaining in the high-stress anomaly photos are heavily fouled and non-reproductive (**c**).

Like growth, reproductive development declined with increasing stress anomalies (Fig. 3c, Table 2), but showed no response to the maximum temperatures (Fig. 3d, Table 2). Receptacles developed on 50–95% of individuals experiencing stress anomalies<2.5 °C, compared with 15–43% of individuals when stress anomalies exceeded 2.5 °C. Moreover, receptacle development in treatments exceeding 2.5 °C occurred before the onset of extreme stress anomalies. By the onset of the reproductive season (May 2013), following peak temperatures, remaining receptacles were heavily fouled and non-reproductive (Fig. 4), and by peak reproductive season (September 2013) no receptacles remained. In contrast, where stress anomalies remained <2.5 °C, receptacles were healthy in May and by peak reproductive season, 75.0±7.3% of individuals were fertile. *Scytothalia* develop and hold their receptacles all year round, but do not successfully reproduce until the coolest months of the year (June–October), because of the low thermal tolerance of pre-settlement stages^22^. Slight changes in phenology as a result of delayed adult development, therefore could push germling development out of the winter reproductive window.

### Tissue health

The decline in growth and reproductive development under high-stress anomalies coincided with an increase in putative disease symptoms and epiphytic fouling (Figs 3e and 4). Tissue health was significantly higher under low-stress anomalies, with 70–100% of thalli remaining free from disease symptoms and epiphytes compared with <50% of healthy tissue on thalli, where stress anomalies exceeded 2.5 °C (Fig. 3e, Table 2). In contrast, tissue health displayed no response to differences in the maximum temperatures (Fig. 3f, Table 2).

## Discussion

Understanding spatial variability in species sensitivity to warming is a fundamental challenge of climate change ecology. Our results demonstrate that both central and rear-edge populations of *Scytothalia* exhibit different absolute temperature tolerances, but have similar thermal-safety margins among populations (that is, Fig. 1b). *Scytothalia's* warm RE contraction, therefore, occurred as a result of high-stress anomalies, the magnitude of which was not observed elsewhere in *Scytothalia's* geographical range. This finding fundamentally differs from the inference that range contractions result from narrow thermal-safety margins in rear-edge populations, in comparison with central populations^1^,^3^,^21^ (that is, Fig. 1a). Importantly, the high thermal-stress anomalies and resultant impacts observed here were consistent across six reefs distributed between two locations separated by over 200 km, but experiencing the same maximum summer temperatures. Therefore, local phenomena (cf regional marine climatic patterns) are unlikely to explain these results. Our experimental results were also consistent with observations from the 2011 marine heatwave, where rear-edge populations experiencing stress anomalies >2.5 °C underwent local extinction, whereas central populations that experienced stress anomalies<2.5 °C survived without any discernible impact^21^.

Constant thermal-tolerance limits are often assumed in climate change studies^1^,^3^,^4^,^21^,^23^ and have received some empirical support^24^. Such patterns of thermal tolerance are likely to occur in organisms with high levels of mobility and connectivity among central and rear-edge populations^6^, or in areas with high environmental heterogeneity throughout a species range^24^. Moreover, the models presented here are not necessarily mutually exclusive, and some organisms may display characteristics somewhere in between Fig. 1a,b. Nevertheless the models highlight the range of responses that might be expected by organisms, and for many there is an increasing recognition that thermal tolerance can vary throughout a species range through local adaptation and acclimatization, resulting in divergent fitness and survivorship of central and marginal populations when exposed to the same conditions^9^,^10^,^14^,^25^.

The marked difference in absolute temperature tolerance among central and rear-edge populations demonstrates a potential for local populations to adjust to future warmer conditions. The speed that thermal-tolerance limits can adjust in response to warming is likely to vary among species (for example, with different generation times) and between genetic and non-genetically based variation in thermal-tolerance limits^12^,^13^. Species that are capable of transgenerational acclimatization, for example, may have greater capacity to adjust to current rates of global warming than locally adapted species relying on natural selection^12^. Currently, the adjustment speed for most species remains unclear and indeed for extreme events such as the marine heatwave observed here, thermal-tolerance adjustment will not be possible. The short duration and extreme impact of the 2011 marine heatwave on the FRE population, and the single generation examined in the translocation experiment is consistent with both genetic (that is, adaptation) and non-genetic (that is, transgenerational acclimatization) processes^13^. For locally adapted species with limited dispersal ability, adaptive management strategies could include targeted translocations of warm adapted genotypes into cooler areas, to boost the resistance of cool adapted populations in areas where warming poses an imminent threat.

The narrow thermal-safety margins observed in this study may reflect the remarkably stable climatic history in South-Western Australia. Since the formation of the Leeuwin Current during the Eocene^26^, organisms in the region have evolved under stable climatic conditions, which today provides an annual temperature range of 6–7 °C in any given location and a highly structured temperature gradient along the coast^27^. The stable latitudinal climatic gradient of South-Western Australia makes it an ideal natural laboratory for comparative studies of this nature, but does not preclude similar responses from occurring in more variable systems. In many coastal environments, synergistic physical processes can result in a mosaic of local climatic conditions with strong implications for the temperature sensitivity of organisms^28^,^29^. Climatic variability, for instance can influence the thermal-tolerance breadth and thermal-safety margins of organisms and is likely to be an important component of locally adapted responses to warming^15^,^16^ (Supplementary Fig. 1). Similarly, variation in genetic diversity may also influence resilience to warming within populations, whereby high-diversity populations have a broader suite of stress-mitigation responses than low-diversity populations^30^.

An important challenge for climate change research will be to find generalities about the nature of thermal-safety margins among and within populations, taxa and systems, to enable a more precise understanding of climate change impacts and identify the areas of greatest vulnerability. We provide a conceptual framework to help address these challenges and demonstrate that central and rear-edge populations can be equally vulnerable to temperature perturbations, in contrast to the assumption underpinning many current climate change impact predictions. Our findings highlight that we may currently be underestimating the vulnerability of central populations to warming, particularly in light of increasing extremes, and/or overestimating the sensitivity of rear-edge populations.

## Methods

### Study location

Translocations took place among Hamelin Bay (34°S, Ce) to Marmion (32°S, RE) and Jurien Bay (30°S, FRE) in the western region of Australia's Great Southern Reef^18^. Seawater temperatures in the region are strongly influenced by the warm, poleward flowing Leeuwin Current and range between summer maximums of 22–24 °C and winter minimums of 17–19 °C for Hamelin Bay and Jurien Bay, respectively (Fig. 2). Because of the directional flow of the current, upwelling is suppressed, resulting in relatively oligotrophic conditions^31^ and a gradual temperature gradient of 1–3 °C (ref. 27).

Within each location three wave exposed sites between 9–12 m depth were selected, each separated by at least 1.8 km. All sites were topographically flat limestone reefs, with mixed canopies dominated by *Ecklonia radiata, Sargassum* spp. (subgen. *Arthrophycus*) and *Scytothalia*, except in Jurien Bay, where *Scytothalia* has been absent since the 2011 marine heatwave^21^. Jurien Bay sites were chosen that had at least 15% *Scytothalia* cover-up until 2011 (ref. 32). Within each translocation site, 10 × 0.25 m^2^ steel mesh quadrants (five mesh per translocation treatment) were fixed to the reef. All mesh were separated by at least 4 m and situated within the canopy, to maximize the survival and growth of the young *Scytothalia*^33^,^34^.

### Translocation experiment set up

Translocations were carried out during the period of coolest seawater temperatures (August and September 2012), to minimize the translocation shock on the seaweeds. Translocations were performed from Ce to RE, Ce to FRE, RE to FRE and procedural control sites were set up among the three RE and within the three Ce sites using self-transplants and naturally attached *Scytothalia,* respectively. Within each source site, 30 young *Scytothalia* (<40 cm total length, with holdfast and no receptacles) were chiselled from the reef ensuring a fragment of limestone was retained beneath the holdfast. For each translocation, individuals were collected from three source sites, kept in buckets of aerated seawater during the day and then transported to their destination in cool, damp calico bags and stored in the dark. Deployments within each destination were staggered so that seaweed from each site spent no longer than 24 h in transit before being returned to the reef. Each individual was measured (total length (*L*) and the maximum circumference (*C*)) and labelled. Within each translocation site, six individuals were transferred to each of the five replicate mesh per treatment using latex-covered cable ties^35^.

Because of logistical constraints natural populations of *Scytothalia* were measured in Hamelin Bay instead of self-transplants. Handling effects were highly unlikely to have been the cause of the observations for three main reasons. First, self-transplants, that is, transplants with the same source and recipient location that otherwise underwent the same handling procedures (including 24 h out of the water) were conducted in Marmion. Both Marmion and Hamelin Bay controls experienced low-temperature stress throughout the translocation experiment and both locations demonstrated the same results in terms of growth, reproductive development and tissue health. The consistency in results between low-anomaly sites that used natural populations and self-translocated population indicates that handling effects did not influence the observed temperature-induced results. Second, the translocation experiment was conducted over 12 months. In the first month of the experiment, some of the translocated seaweeds lost biomass, most likely as a result of handling effects (Supplementary Fig. 2). After the initial handling effects, all populations including the natural and translocated populations gained biomass up until the onset of warm summer temperatures (Supplementary Fig. 2). Moreover, receptacles grew and disease symptoms were low on all controls and treatments up until the warm summer months. These results clearly indicate that while there may have been some initial handling effects, these effects were short term and occurred before the onset of the warm summer months when the temperature differences were observed. Finally, habitat conditions of the translocated populations and natural populations were the same, with the exception of temperature. All individuals tracked throughout the experiment were tagged, labelled and grown inside kelp (*E. radiata*) dominated canopies of densities between 8 and 12 sporophytes per m^2^, described above.

### Growth measurements

All labelled specimens were measured every 1–2 months at all sites. Total length (*L*) and circumference (*C*) were converted to fresh weight (*W*_F_) by *W*_F_=*LC*^2^ (following refs 33, 36; Supplementary Fig. 3). Growth rates were calculated as the change in individual biomass between successive sampling times for each replicate specimen. Change in biomass for each growth period was standardized to grams fresh weight per 30 days. The first sampling period after the translocation was removed from the analysis to avoid any influence from short-term stress and transplantation shock. Reproductive development was determined by recording presence/absence of receptacles on each individual. Tissue health of individuals was assessed *in situ* on the lower middle and upper sections of the thallus by recording per cent cover of putative disease symptoms including thallus bleaching and/or dark brown spots and visible epibiosis^33^.

### Thermal-stress anomalies

Thermal-stress anomalies^17^ experienced by each transplant and control population, were calculated for each 1–2 month growth period by comparing the mean daily temperature in the recipient location to the long-term mean summer maximum (averaged from 2006 to 2013) in the seaweeds location of origin. ‘Maximum stress anomalies' refer to the highest stress anomalies recorded throughout the experiment or heatwave. While stress anomalies are usually calculated from a single location, the nature of space-for-time substitution experiments required temperatures from source and recipient locations to be used. Mean daily temperatures were calculated from hourly temperature measurements recorded 5 cm above the reef (9–12 m depth) at three reefs within each location using ‘TidbiT' loggers^27^ (Onset Stowaway logger, model TBI32-05+37, accuracy±0.2 °C). Degree heating weeks (DHW) were calculated to assess the importance of duration of stress anomalies^37^. DHW sum-up temperatures exceeding a 1 °C stress anomaly and standardize the number of weekly measurements throughout any given 12 week period using the equation: DHW=0.14*(sum of daily temperatures exceeding 1 °C throughout 12 week period).

### Statistical analyses

The relationships between physiological performance metrics (growth, reproductive development and tissue health) and temperature metrics (thermal-stress anomaly and maximum temperature) were tested using linear mixed-effects models. Separate nested intercept models were used to test the relationship between each performance and temperature metric combination. The relationship between growth rates and each temperature metric (stress anomalies and temperature) was first analysed across the entire year to assess growth responses across a broad range of temperatures and stress anomalies. Analyses were structured so that ‘time' (*n*=5, continuous random effect) represented repeated bi-monthly measures of mean seaweed growth within each mesh (*n*=75), site and location combination^38^. Growth locations and sites nested within growth locations were used as random grouping variables to account for the nested structure of the design and local growing conditions.

In addition, mean summer (January–March) growth rates at each treatment/site combination were analysed in response to stress anomalies and maximum temperatures to the test growth responses of *Scytothalia* during the warmest part of the year (Fig. 2). The relationship between both reproductive development and tissue health and the two temperature metrics was analysed using the treatment and site means (*n*=15) measured in March–May 2013 following the warmest temperatures (Fig. 2) and corresponding to the beginning of the winter reproductive season^22^. For each of these analyses, growth location (Hamelin Bay, Marmion or Jurien Bay) was used as a random grouping variable to account for nested structure of the experimental design and local growing conditions. The temperature metric that best predicted each of the seaweed response variables was determined using Akaikes information criterion (AIC), by selecting the model which displayed the lowest AIC value. Assumptions for the linear mixed-effects models were checked by examining the normalized residual plots for homogeneity of variance. Analyses were performed using the nlme package of R statistical software (Version 3.01, R_Development_Core_Team 2013).

## Additional information

**How to cite this article:** Bennett, S. *et al.* Central and rear edge populations can be equally vulnerable to warming. *Nat. Commun.* 6:10280 doi: 10.1038/ncomms10280 (2015).

## Supplementary Material

Supplementary Information Supplementary Figures 1-3 and Supplementary References.

## Figures and Tables

**Table 1:** Compilation of temperature impacts experienced by rear-edge and central populations of *Scytothalia* during the heatwave and translocation experiment.

**Source location**	**Heatwave/experimental treatment**	**Max temperature (°C)**	**Max stess anomaly (°C)**	**Max DHW (°C-weeks)**	**Recorded impact**
Central	2011 Heatwave	23.86	2.12	8.83	None
(Ce, Hamelin Bay)	Ce to Ce	23.20	1.46	0.70	None
	Ce to RE	24.41	2.67	22.54	Net biomass loss, failed reproductive development, >60% cover of putative disease symptoms and mortality
	Ce to FRE	24.40	2.66	22.87	Net biomass loss, failed reproductive development, >50% cover of putative disease symptoms and mortality
Range edge	2011 Heatwave	25.38	1.91	3.69	None
(RE, Marmion)	RE to RE	24.41	0.95	0	None
	RE to FRE	24.40	0.93	0	None
Former range edge (FRE, Jurien Bay)	2011 Heatwave	27.34	3.50	9.46	100% Mortality

Ce, central; FRE, former warm rear edge; RE, rear edge.

Impacts were compared for three common temperature stress metrics: maximum temperature; maximum thermal-stress anomaly; and maximum degree heating weeks (Max DHW). Note that only stress anomalies >2.5 °C consistently correspond impact among the different locations.

**Table 2:** Mixed-effects model summaries for *Scytothalia* performance metrics in response to thermal-stress anomalies and maximum temperatures.

**Fixed effects**						**Random effects**		
**Response variable**	**Effect**	**coef.**	**s.e.m.**	***t*****-value**	***P*****-value**	**AIC**	**Spatial scale (no. observations)**	**Within group variance**
Growth (all seasons)	**Stress anomaly**	**−4.65**	**1.13**	**−4.09**	**<0.001**	**2,454.53**	Time|location (3)	<0.001
							Time|site/location (9)	<0.001
							Residual	3.33
	Max temp	−4.51	1.27	−3.53	<0.001	2,458.29	Time|location (3)	<0.001
							Time|site/location (9)	<0.001
							Residual	3.36
Growth (summer)	**Stress anomaly**	**−16.41**	**5.94**	**−2.76**	**0.024**	**97.07**	Location (3)	5.11
							Residual	16.10
	Max temp	0.12	10.37	0.012	0.992	102.00	Location (3)	3.98
							Residual	21.58
Reproductive development	**Stress anomaly**	**−0.31**	**0.05**	**−5.86**	**<0.001**	**5.63**	Location (3)	0.16
							Residual	0.16
	Max temp	0.09	0.20	0.45	0.729	19.31	Location (3)	0.11
							Residual	0.31
Tissue health	**Stress anomaly**	**−29.14**	**3.85**	**−7.55**	**<0.001**	**116.17**	Location (3)	9.03
							Residual	11.57
	Max temp	−19.92	14.43	−1.38	0.399	134.48	Location (3)	0.003
							Residual	26.94

AIC, Akaikes information criterion.

Bold rows indicate the best fit model (based on lowest AIC) between the two temperature metrics, for each of the three performance indices. Growth location was used as a random grouping variable.
