# Supplementary material for: Central and rear-edge populations can be equally vulnerable to warming
Source: Nat Commun. 2015 Dec 22;6:10280. doi: 10.1038/ncomms10280 (PMC4703895; doi:10.1038/ncomms10280)
Supplement: Supplementary Information — Supplementary Figures 1-3 and Supplementary References. [file ncomms10280-s1.pdf]

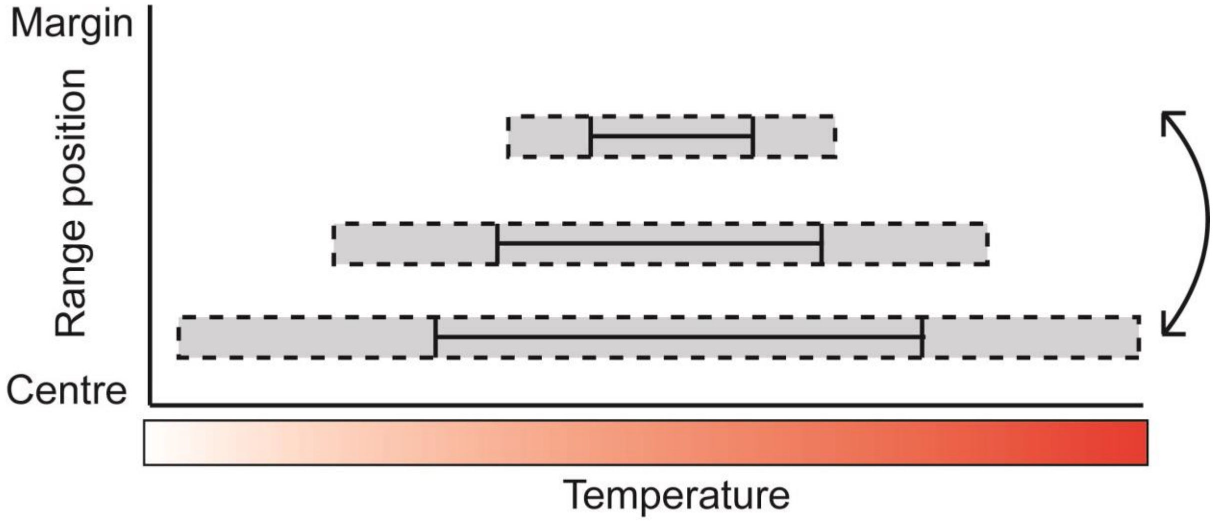

Supplementary Figure 1. Conceptual diagram illustrating a third possible relationship between a populations experienced temperature breadth (solid line) and its temperature tolerance breadth (dashed box), throughout a species geographical range. Distance between the solid line and the edge of the dotted line represents thermal safety margin. Experienced climatic ranges differ in absolute values and variability among populations. Thermal tolerance breadth and thermal safety margins differ among populations in relation to experienced climatic variability. Arrow indicates that order of tolerance range with respect to range position is arbitrary.

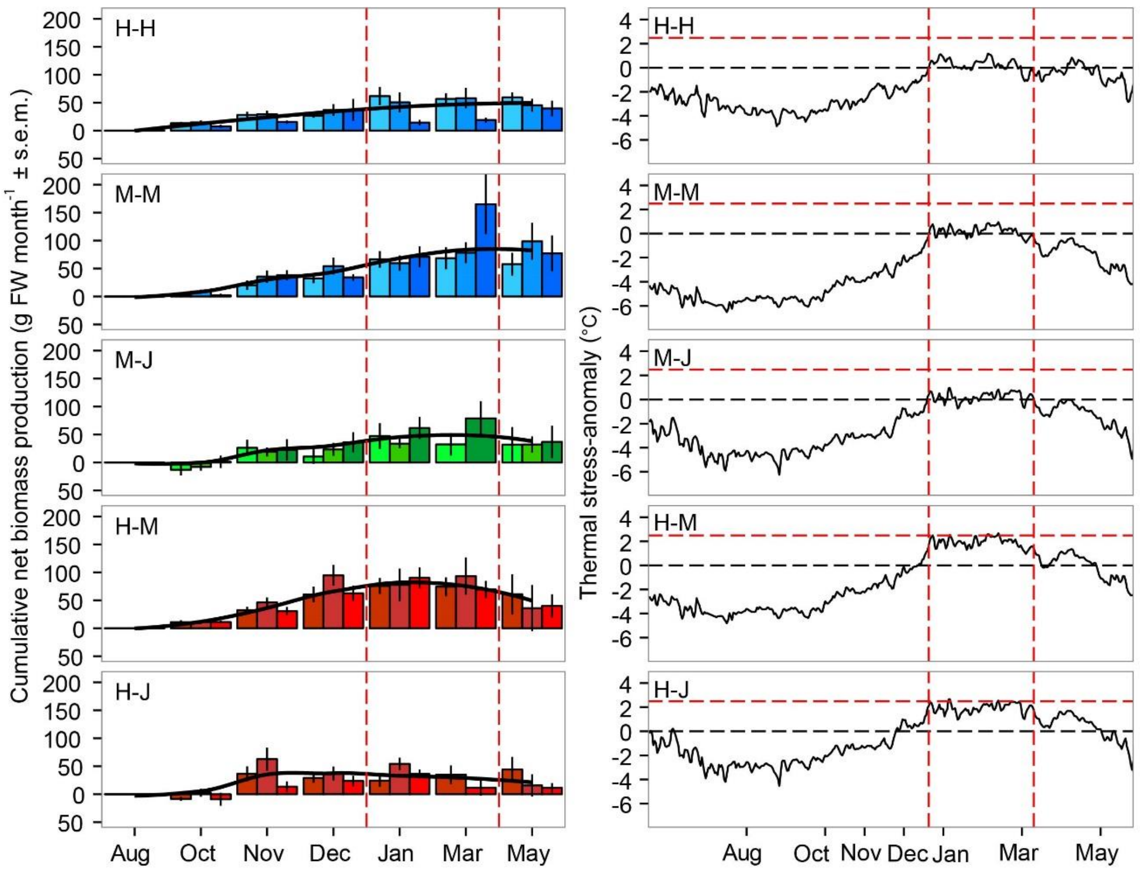

Supplementary Figure 2. **Cumulative net biomass changes and thermal stress-anomalies experienced by *Scytothalia dorycarpa* during the translocation experiment**. Left hand series of panels illustrates the mean biomass accumulation per individual relative to initial conditions (bars) and the overall population growth trajectory (black line) over the course of the experiment. Different vertical panels represent the different translocation treatments whereby Ce-Ce = central to central, RE-RE = rear edge to rear edge, RE-FRE = rear edge to former rear edge, Ce-RE = central to rear edge, and Ce-FRE= central to former rear edge. The three different colour shades within each vertical panel represent replicate sites. Right hand series of panels illustrates the thermal stress-anomalies experienced by the transplants throughout the experiment, relative to their reef of origin. Vertical dashed bars correspond to left hand panels and demarcate the warmest summer period. Horizontal dashed bars demarcate stress-anomalies of 0ºC (black) and 2.5ºC (red).

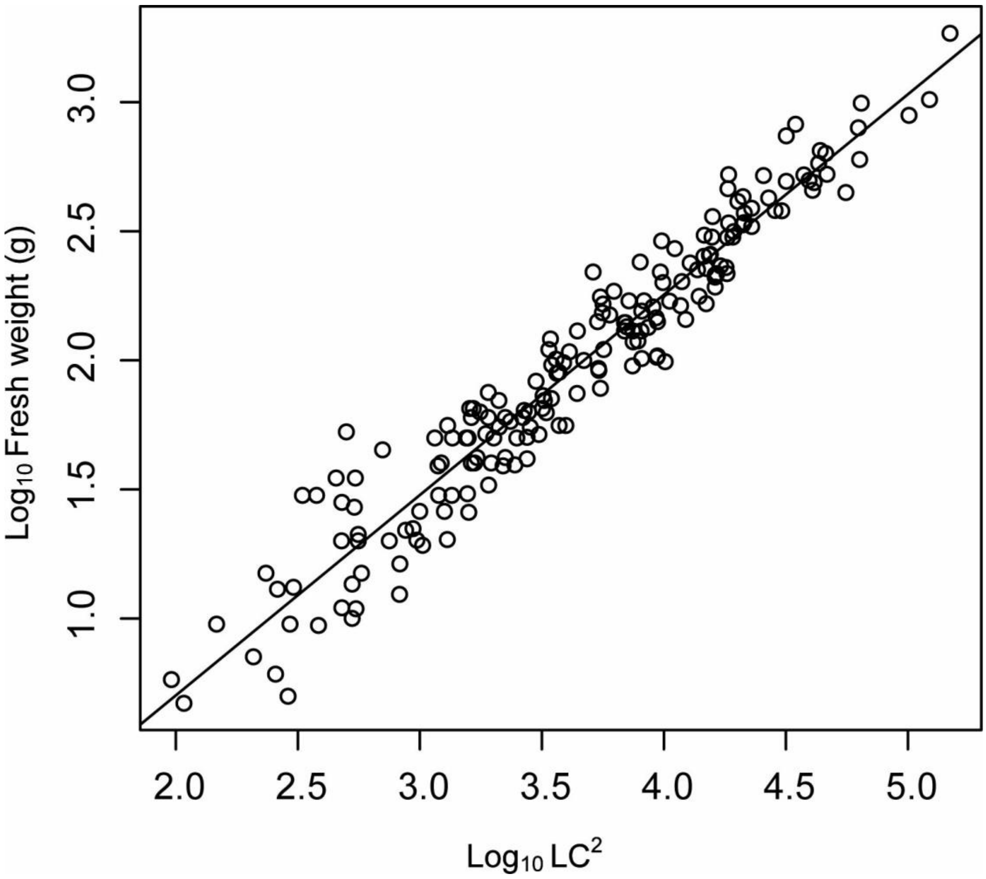

Supplementary Figure 3. **Significant relationship between *Scytothalia dorycarpa* fresh weight and LC^2 ($F_{1,187} = 2536$, $p < 0.001$, adjusted $R^2 = 0.931$).** L = length, and C = maximum circumference of the seaweed [1].

References

1. Aberg P. Measuring size and choosing category size for a transition matrix study of the seaweed *Ascophyllum nodosum*. *Marine Ecology Progress Series* **63**, 281-287 (1990).
